# Center of Pressure of Medial Knee Contact Force Predicts Future Transition Risk of Knee Surgery in Patients with Knee Osteoarthritis

**DOI:** 10.1007/s10439-024-03664-0

**Published:** 2025-01-31

**Authors:** Yamagata Momoko, Taniguchi Masashi, Tateuchi Hiroshige, Motomura Yoshiki, Kobayashi Masashi, Ichihashi Noriaki

**Affiliations:** 1 Faculty of Rehabilitation, Kansai Medical University, 18-89 Uyama Higashimachi, Hirakata, Osaka 573-1136 Japan; 2 Human Health Science, Graduate School of Medicine, Kyoto University, Kyoto, Japan; 3 Kobayashi Orthopaedic Clinic, Kyoto, Japan

**Keywords:** Knee osteoarthritis, Knee surgery, Gait, Knee contact force

## Abstract

**Purpose:**

This study aimed to explore whether mechanical load features, including the magnitude of the medial knee contact force (KCFmed) and its center of pressure (KCFcop) during gait, predict future surgery in patients with knee osteoarthritis (OA).

**Methods:**

Twenty-six patients with knee OA walked three times at a comfortable speed, and the external knee adduction moment (KAM), flexion moment (KFM), and total knee moment of the KAM and KFM (KTM) were assessed. We further evaluated KCFmed and KCFcop using a musculoskeletal model. The values of knee moments and KCFmed were extracted at the first and second peaks, and the average KCFcop location and amount of KCFcop displacement were calculated during the early-, mid-, and late-stance phases. Ten years after data collection, we confirmed whether the patients required knee surgery (Surg_OA) or not (NonSurg_OA).

**Results:**

Twenty-four patients with complete data were divided into Surg_OA and NonSurg_OA groups. The Surg_OA group had significantly lower KTM, KFM, and KCFmed values at the first peak than the NonSurg_OA group. In the Surg_OA group, KCFcop shifted toward the joint center during the mid- and late-stance phases, and the amount of KCFcop displacement was small during the mid-stance phase. No significant differences were observed in the other parameters.

**Conclusion:**

Our findings demonstrated that individuals who underwent knee surgery within 10 years showed suppressed KCFmed magnitudes in the first half of the stance phase, whereas they received sustained force on a localized area of the medial compartment during the mid-stance phase.

## Introduction

Knee osteoarthritis (OA) is a common disease that leads to knee pain, and the primary treatments are conservative and surgical therapies. In many cases, knee OA is initially treated conservatively, but surgical therapy is considered if symptoms (including knee pain) persist or deteriorate or OA progresses after conservative therapy [1]. However, surgical therapy is accompanied by the risk of complications such as postoperative pain and discomfort [2–4]. Therefore, identifying the factors that cause severe pain and OA progression at an early stage is crucial for preventing surgery.

Repeated knee contact force (KCF) in daily life exacerbates pain and the progression of OA. As noninvasive KCF measurement is difficult, the external knee adduction moment (KAM) and flexion moment (KFM) have been widely used as alternative measures of KCF in the medial compartment (KCFmed) [5–8]. Additionally, the usefulness of the total knee moment of the KAM and KFM (KTM) has also been shown in previous studies [8, 9]. Such studies have shown that the KTM accurately reflects the measured KCFmed better than the KAM or KFM [9] and is sensitive to different symptoms and OA progression in patients with knee OA [8].

In contrast, unlike knee joint moments, musculoskeletal models allow the estimation of KCFmed by considering factors such as muscle contraction [10–12]. These techniques have been applied to patients with knee OA and have been used to compare symptom severity [10] and gait modifications [11]. Moreover, the center of pressure in KCFmed (KCFcop) would provide additional information about the mechanical load applied to knee joint. During walking, KCFmed is generated over the entire contact surface of the medial compartment, whereas KCFcop is a quantitative measure of the localized force in the medial compartment. For example, high joint stiffness induced by muscle coactivation around the knee joint is a gait characteristic often observed in patients with knee OA and is considered a gait strategy to stabilize the knee joint and avoid knee pain [13–15]. However, a previous study on patients with knee implants found that muscle coactivation of the quadriceps and gastrocnemius increased the KCF by up to 1 body weight in some patients [16], and increased joint stiffness due to muscle coactivation has been reportedly associated with decreased articular cartilage [17]. Particularly, damage to the articular cartilage is prompted by the coactivation of the medial muscles relative to the lateral muscles of the knee joint [17]; therefore, localized loads specific to a certain area on the medial compartment may increase the risk of cartilage damage and surgical therapy. However, it is unclear how KCF is applied to the localized area of the medial compartment during gait in patients with knee OA and which indices of mechanical properties determine whether patients require surgery in the future.

This exploratory study aimed to clarify the features of mechanical load (i.e., knee joint moments, KCFmed, and KCFcop) during gait in patients with knee OA who will undergo surgery in the future. We expected that patients with knee OA who will undergo surgery would have larger KCFmed and knee joint moments than those who will not undergo surgery, and that KCFcop in these patients would be concentrated in a localized area.

## Materials and Methods

### Participants

Twenty-six female patients with knee OA (mean ± standard deviation [SD]; age 63 ± 8 years, body mass 56.5 ± 5.4 kg, height 1.6 ± 0.1 m) participated at the baseline. We included participants with unilateral or bilateral knee OA (i.e., radiographic Kellgren and Lawrence [K/L] grade ≥ 1) who could walk without an assistive device and who were not scheduled for knee surgery at baseline. The exclusion criteria were any surgery on both lower limbs at baseline and neurological or balance disorders requiring assistive devices. All the participants were informed of the experimental procedures and provided written informed consent. This study was approved by the Ethics Committee of Kyoto University Graduate School and Faculty of Medicine (R1647).

At baseline, self-reported symptom and function scores were evaluated using the Japanese Knee Osteoarthritis Measure to assess knee pain severity and physical function level [18]. The symptom scores ranged from 0 (no pain) to 10 (worst pain ever), and the function scores ranged from 0 (no physical dysfunction) to 100 (worst physical dysfunction).

Ten years after baseline, the participants were divided into patients who underwent knee surgery (Surg_OA) and those who did not (NonSurg_OA) based on the medical records. Two participants were excluded due to missing data, and the remaining patients with knee OA were divided into NonSurg_OA (*n* = 16) and Surg_OA (*n* = 8) groups.

### Gait Analysis at Baseline

Participants were asked to walk on a 6-m walkway at their preferred speed. After the reflective markers were placed according to the Vicon Plug-in-Gait full-body model marker placement protocol, three successful trials were used for further analysis. Kinematic data were collected using a motion capture system (Vicon, Oxford, UK) at a sampling rate of 100 Hz, and marker trajectories were filtered using a 4th-order low-pass Butterworth filter at 6 Hz. Ground reaction force data were collected using two force plates (Kistler, Winterthur, Switzerland) at a sampling rate of 1000 Hz, and the signals were filtered using a 4th-order low-pass Butterworth filter at 20 Hz.

In addition to gait speed, we evaluated the following measures of the more affected side to characterize the gait pattern: KAM, KFM, and KTM at the first and second peaks, vertical ground reaction force (GRF) at the first and second peaks, and peak sagittal and frontal angles of the knee, hip, and ankle joints. KAM and KFM were calculated using an inverse dynamics approach that solves for the knee’s composite moment using the Newton–Euler equations [19]. The magnitudes of the knee moment are mainly altered by the magnitude of the GRF and the length of the lever arm (i.e., the distance between the frontal or sagittal-ground reaction force and the knee joint center) [20]. KTM was computed as the sum of KAM and KFM, the external moments acting in the frontal and sagittal planes, respectively [9]. For further statistical comparisons, these measures were averaged across three trials.

### Musculoskeletal Model

A detailed description of the musculoskeletal model has been provided in a previous study [10]. To briefly summarize, the musculoskeletal model was created using the Twente Lower Extremity Model version 2 (TLEM2) in the AnyBody Modeling System v.7.1 (AnyBody, Aalborg, Denmark). The model contained 11 segments (pelvis, both sides of the femurs, patellas, shanks, talus, and feet) and eight joints (hip [3DoFs], knee [3 DoFs], talocrural [1DoF], and subtalar joints [1 DoF]). Each lower limb contained 55 muscles, with 169 elements modeled using Hill’s model.

To create a participant-specific model, a scaled musculoskeletal model based on anthropometric data [21] was adjusted using the femorotibial angle (FTA) for each participant, and the lengths and widths of the segments were optimized according to the marker data. Acquired data (e.g., marker trajectories and GRF) were input to participant-specific models for inverse dynamics analysis. In this process, a numerical optimization procedure with a 3rd-order polynomial muscle recruitment criterion was applied, and the total KCF was estimated. The total KCF was decomposed into forces on the medial compartment using 12 nodes (three in the mediolateral direction and four in the anteroposterior direction) on the scaled medial condyles of the tibia [10]. Nodes are specific points that define the location on the medial condyle of the tibia within a musculoskeletal model. The KCFmed was computed as the sum of the knee contact forces on the medial compartment, and the values at the first and second peaks were extracted and averaged across the trials for further analyses. Additionally, we calculated the mediolateral and anteroposterior KCFcop locations on the medial compartment relative to the center of the knee joint, and the averaged two-dimensional locations and displacements were computed during the early- (period from foot contact to first-peak KCFmed), mid- (period from first-peak KCFmed to second-peak KCFmed), and late- (period from second-peak KCFmed to toe-off) stance phases.

### Statistical Analysis

All statistical analyses were performed using SPSS version 26 (IBM Corp., Armonk, NY, USA). The significance level was set at *p* = 0.05. To test group-related differences in gait parameters (i.e., gait speed, GRFs, joint angles, and moments [KAM, KFM, and KTM], KCFmed, and KCFcop), Mann–Whitney *U* tests were performed to compare the measures between NonSurg_OA and Surg_OA. We used non-parametric procedures because some measures deviated from the normal distribution with the Kolmogorov–Smirnov test, and the number of participants in the Surg_OA group was relatively small.

## Results

Table 1 presents the physical characteristics of the participants. Significant differences between the NonSurg_OA and Surg_OA groups were found in the FTA and function scores; the Surg_OA group displayed a larger FTA (*p* < 0.05) and worse physical function than the NonSurg_OA group (*p* < 0.05). Regarding gait parameters, we found a significantly lower first-peak GRF (*p* < 0.05), larger knee adduction angle (*p* < 0.05), and lower hip adduction angle (*p* < 0.05) in the Surg_OA group than in the NonSurg_OA group (Table 2). No significant differences were found in other measures.

**Table 1:** Description of participants

	NonSurg_OA (*n* = 16)	Surg_OA (*n* = 8)	Effect size
Age (years)	63 (7)	65 (8)	–
Height (m)	1.6 (0.0)	1.5 (0.1)	–
Weight (kg)	57.7 (5.8)	55.7 (4.5)	–
K/L grade	I: 2, II: 9, III: 2, IV: 3	II: 1, III: 2, IV: 5	–
FTA*	178.6 (3.1)	181.0 (3.0)	0.44
Function Score*	15.8 (11.0)	24.0 (6.5)	0.41
Symptom Score	2.8 (3.1)	3.4 (2.3)	–
Surgical Method	–	TKA: 5 UKA: 1 HTO: 2	

The values are mean (SD). *Significant differences between knee OA patients without any surgery (NonSurg_OA) and those with surgery (Surg_OA) are shown with asterisk (*p* < 0.05). Function and symptom scores were assessed by the Japanese Knee Osteoarthritis Measure.

*NonSurg_OA* patients who did not undergo surgery, *Surg_OA* patients who underwent knee surgeries, *K/L grade* Kellgren and Lawrence Grade, *FTA* Femoro-tibial angle, *TKA* Total Knee Arthroplasty, *UKA* Unicompartmental Knee Arthroplasty, *HTO* High Tibial Osteotomy

**Table 2:** Gait parameters

	NonSurg_OA (*n* = 16)	Surg_OA (*n* = 8)	Effect size
Gait Speed (m/s)	1.2 (0.2)	1.2 (0.1)	–
First-peak GRF (N) *	652.6 (60.5)	594.6 (53.3)	0.43
Second-peak GRF (N)	605.0 (61.8)	588.6 (46.4)	–
Knee Flexion Angle (°)	20.2 (7.2)	16.3 (4.9)	–
Knee Adduction Angle (°) *	5.0 (6.0)	8.4 (2.1)	0.48
Hip Flexion Angle (°)	30.9 (8.8)	35.7 (4.2)	–
Hip Adduction Angle (°) *	8.7 (4.2)	4.8 (2.7)	0.46
Ankle Dorsiflexion Angle (°)	7.6 (5.6)	5.0 (5.2)	–
Ankle eversion Angle (°)	2.0 (1.7)	2.6 (2.0)	–

The values are mean (SD). *Significant differences between knee OA patients without any surgery (NonSurg_OA) and those with surgery (Surg_OA) are shown with asterisk (*p* < 0.05).

*NonSurg_OA* patients who did not undergo surgery, *Surg_OA* patients who underwent knee surgeries, *GRF* Ground Reaction Force

**Fig. 1:**
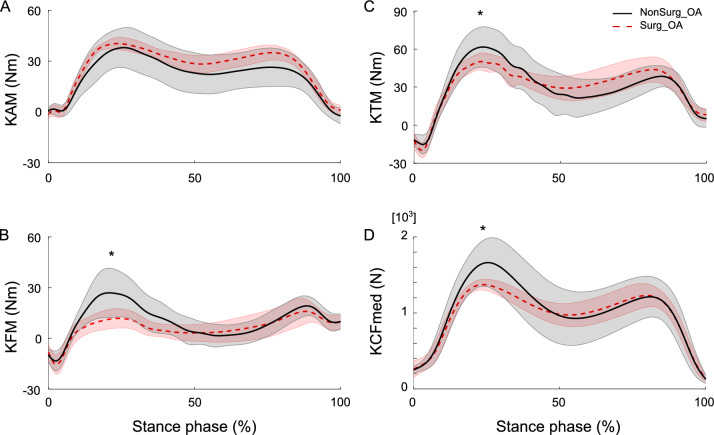
Time profiles of knee moments and KCFmed cross-participant mean with standard deviation. The black solid and red dotted lines represent the average KAM (panel **A**), KFM (panel **B**), KTM (panel **C**), and KCFmed (panel **D**) values for the NonSurg_OA and Surg_OA groups, respectively. Significant differences between Surg_ and NonSurg_OA are indicated with one star (*p* < 0.05). *KCFmed* knee contact force on the medial compartment, *KAM* external knee adduction moment, *KFM* external knee flexion moment, *KTM* knee total moment, *NonSurg_OA* patients who did not undergo surgery, *Surg_OA* patients who underwent knee surgery

The results of the between-group comparisons of the knee joint moments (KAM, KFM, and KTM) and KCFmed are shown in Fig. 1. We found no significant differences in the first- and second-peak KAM, whereas significant differences were found in the first-peak values of KFM, KTM, and KCFmed; the values in the Surg_OA group were significantly lower than those in the NonSurg_OA group (*p* < 0.05).

**Fig. 2:**
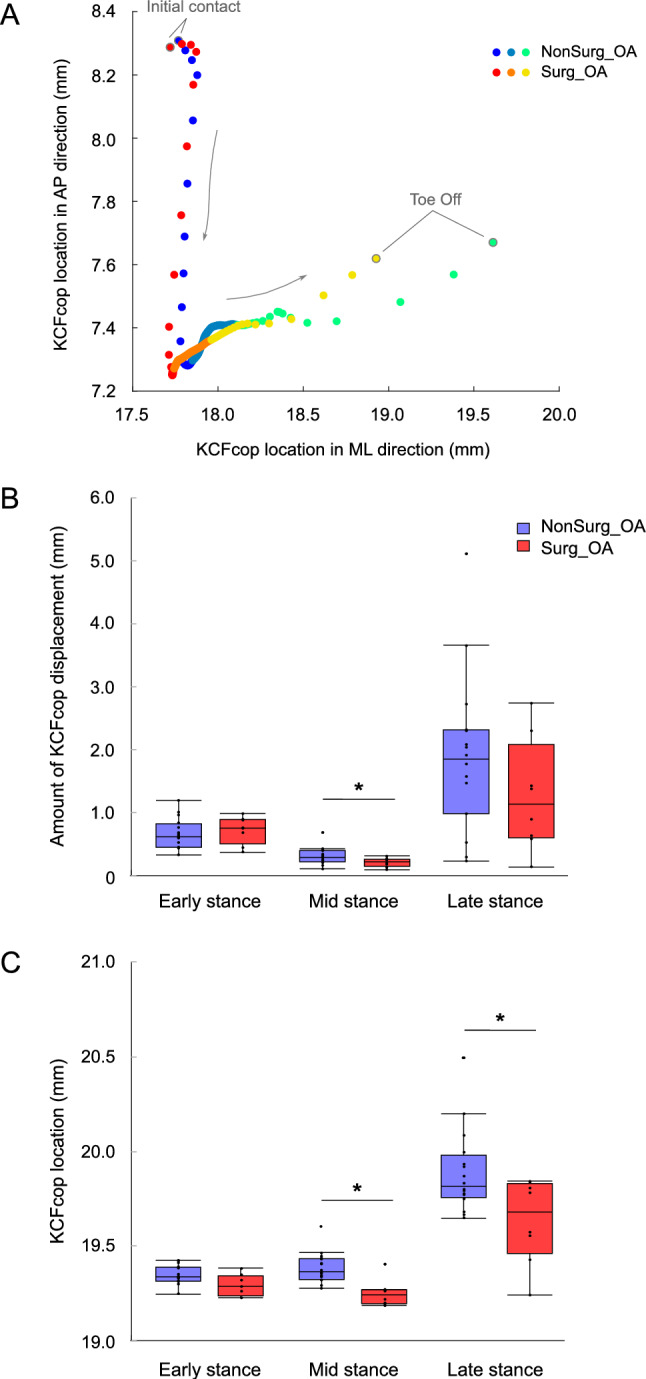
Time profiles and box plots of KCFcop. **A:** Time profiles of KCFcop from the initial contact to toe-off. The bluish and reddish colors represent the KCF cop locations in NonSurg_OA and Surg_OA groups, respectively. The blue and red dots are the average KCFcop locations in the early stance phase, the light blue and orange dots are those in the mid-stance phase, and the green and yellow dots are those in the late-stance phase. **B** and **C:** Box plots of the amount of KCFcop displacement and KCFcop location in NonSurg_OA (blue boxes) and Surg_OA (red boxes) during the early-, mid-, and late-stance phases. Dots represent the values for each participant. Significant differences between Surg_ and NonSurg_OA are indicated with one star (*p* < 0.05). *KCFcop* center of pressure in knee contact force, *NonSurg_OA* patients who did not undergo surgery, *Surg_OA* patients who underwent knee surgery

Figure 2 shows the KCFcop indices. We found significant differences in the two-dimensional location of KCFcop between the Surg_OA and NonSurg_OA groups during the mid- and late-stance phases, and the location of KCFcop in the Surg_OA group was closer to the center of the knee joint (*p* < 0.05). Additionally, during the mid-stance phase, the two-dimensional KCFcop displacement was significantly smaller in the Surg_OA group than in the NonSurg_OA group (*p* < 0.05).

## Discussion

We aimed to clarify the features of mechanical load during gait in patients with knee OA deemed to be at a high risk of requiring surgery in the future. The first-peak KFM, KTM, and KCFmed values in patients who underwent surgery (Surg_OA) were significantly lower than those in patients who did not (NonSurg_OA). However, this difference was not observed for the KAM. We further found that the KCFcop in the Surg_OA group shifted toward the joint center during the mid- and late-stance phases and that the amount of KCFcop displacement decreased during the mid-stance phase. These results partially support our hypothesis that patients with knee OA who will undergo surgery in the future would display high mechanical loads and concentrated KCFcop in a localized region. To our knowledge, this study is the first to evaluate in detail the mechanical characteristics of gait in patients with knee OA who will undergo future surgery using musculoskeletal models.

The Surg_OA group had lower KCFmed, KFM, and KTM values at the first peak than the NonSurg_OA group. In contrast, the KAM in Surg_OA did not increase despite the large FTA and adduction angle during gait. Given the small GRF in the Surg_OA group, patients with knee OA who require surgery may suppress the increase in knee loading owing to a reduction in GRF. Similar gait properties have also been observed in previous studies; first-peak KFM was lower in patients with more severe knee OA [22], and first-peak GRF and KTM were reduced by pain and inflammation in the knee joint [8]. The Surg_OA group is expected to increase the requirement for such compensatory patterns because its kinematic environment (i.e., the large adduction angle of the knee joint) increases the risk of large knee loading.

Additionally, the Surg_OA group had lower adduction angle of the hip joint than the NonSurg_OA group. There is a high possibility that hip dysfunction would affect knee biomechanics [23]. The lower limb joints are interrelated during gait; a small adduction angle of the hip joint (i.e., relative hip abduction motion) in the Surg_OA group may influence the knee adduction angle and knee-loading patterns. To eliminate the need for the compensatory patterns observed in the Surg_OA group and prevent the progression of knee OA, treatments that target the kinetic chain of the lower limb, including the hip joint, may be beneficial rather than only the knee joint.

We also found that the Surg_OA group exhibited a shift in KCFcop toward the center of the knee joint and a decrease in KCFcop displacement during the mid-stance phase. The mid-stance phase is the duration during which the mechanical load is continuously applied, and the decrease in the KCFcop displacement during this period implies that the loadings are concentrated and sustained in a certain area (in the case of Surg_OA, an area close to the center of the knee joint). In other words, although Surg_OA suppressed the magnitude of the mechanical load during the stance phase, it may continuously receive loads in a localized area. A previous study found that the contact point of the medial compartment in patients with knee OA shifted toward the joint center compared with that in healthy adults [24]. Additionally, individuals with lower kinematic stability of the knee joint, such as patients with anterior cruciate ligament defects, have been shown to shift the contact point toward the joint center during squats [25]. There is a high possibility that the Surg_OA group exhibits low knee joint stability due to a large FTA. In participants with low knee joint stability, the contact point might have shifted to the joint center to avoid a large mechanical load on the knee joint owing to the extreme medial or lateral shift of the contact point.

The loading environment of the joint is constrained by several factors, such as its morphology and congruence; particularly, the loading regions of the tibia in patients with knee OA are influenced by alterations in ligament properties [26], muscle strength [27], or muscle activity [15, 28]. For instance, patients with severe knee OA who were scheduled to undergo surgery within a week had greater muscle coactivation of the lower extremities than patients with mild knee OA or asymptomatic controls [28]. The regions that are loaded infrequently (i.e., the low-loading regions) show degenerated cartilage properties compared to the high-loading regions with great thickness and high mechanical properties [29]. Therefore, the shift to infrequently loaded regions due to factors such as muscle coactivation may fail to adapt to loading, likely prompting cartilage degeneration [30]. The previous interpretation that a reduced mechanical load in patients with knee OA is a compensatory strategy for knee pain may have overlooked the localized loads on the medial compartment, and the KCFcop estimated using musculoskeletal models could be a useful index for predicting the need for surgery.

The primary limitations of this study were its small sample size and the imbalanced number of OA grades in the Surg_OA and NonSurg_OA groups. A larger sample size and a balanced number of OA grades are needed to clarify whether the findings obtained in this study can be more broadly applied to all patients with knee OA. Second, all patients in this study were female. Given that there are sex-related differences in walking properties and mechanical loadings of the knee joint [31], different walking properties between the Surg_ and NonSurg_OA groups may have been observed in male patients with knee OA. Third, while total knee arthroplasty is the most common method used in patients with severe pain, this study included several surgical methods. However, because no outliers were found among the surgical methods, we believe that the different surgical methods did not affect our findings. Finally, the musculoskeletal model used in this study did not consider muscle coactivation. Since muscle coactivation, often observed in patients with knee OA, is one of the factors that increase KCFmed, improving the musculoskeletal model may be necessary for patients with knee OA. Resolving these limitations and exploring how the knee-loading patterns observed in Surg_OA influence the long-term health status could provide additional information for understanding the gait patterns inducing the progression of knee OA.

In summary, our results suggest that individuals with reduced mechanical load (i.e., KTM, KFM, and KCFmed) during the first half of the stance to avoid knee pain will undergo knee surgery in the future. Moreover, individuals who will undergo knee surgery in the future have decreased KCFcop displacement in an area close to the joint center; the concentrated and sustained force in this area may increase the risk of requiring future surgery. Detailed measures of KCF can be used to predict potential risks of requiring surgery in the future.

## Citation Diversity Statement

We recognize biases in citation practices, such as papers by women and other minority scholars being cited less than the number of articles, and have worked to ensure that appropriate references are made to articles that include authors of fair gender and race.

## Data Availability

The data used in this study are not available due to ethical restrictions.
