# Correction: Center of Pressure of Medial Knee Contact Force Predicts Future Transition Risk of Knee Surgery in Patients with Knee Osteoarthritis

**DOI:** 10.1007/s10439-025-03707-0

**Published:** 2025-03-09

**Authors:** Momoko Yamagata, Masashi Taniguchi, Hiroshige Tateuchi, Yoshiki Motomura, Masashi Kobayashi, Noriaki Ichihashi

**Affiliations:** 1 Faculty of Rehabilitation, Kansai Medical University, 18-89 Uyama Higashimachi, Hirakata, Osaka 573-1136 Japan; 2 Human Health Science, Graduate School of Medicine, Kyoto University, Kyoto, Japan; 3 Kobayashi Orthopaedic Clinic, Kyoto, Japan

**Correction to: Annals of Biomedical Engineering** 10.1007/s10439-024-03664-0

In the published article the authors' given names and family names are transposed. The correct names are Momoko Yamagata, Masashi Taniguchi, Hiroshige Tateuchi, Yoshiki Motomura, Masashi Kobayashi, and Noriaki Ichihashi.

